# Complex Determinants of Epithelial: Mesenchymal Phenotypic Plasticity in Ovarian Cancer

**DOI:** 10.3390/cancers9080104

**Published:** 2017-08-09

**Authors:** Yuliya Klymenko, Oleg Kim, M. Sharon Stack

**Affiliations:** 1 Department of Chemistry and Biochemistry, Harper Cancer Research Institute, University of Notre Dame, Notre Dame, IN 46617, USA; yklymenk@nd.edu; 2 Medical Sciences Program, Indiana University School of Medicine, Bloomington, IN 47405, USA; 3 Department of Applied and Computational Mathematics and Statistics, Harper Cancer Research Institute, University of Notre Dame, Notre Dame, IN 46617, USA; okim@nd.edu; 4 Department of Mathematics, University of California Riverside, Riverside, CA 92521, USA

**Keywords:** ovarian cancer, intraperitoneal metastasis, cadherins, heterogeneity, epithelial-to-mesenchymal transition (EMT), mesenchymal-to-epithelial transition (MET), intraperitoneal tumor microenvironment, computational modeling of EMT

## Abstract

Unlike most epithelial malignancies which metastasize hematogenously, metastasis of epithelial ovarian cancer (EOC) occurs primarily via transcoelomic dissemination, characterized by exfoliation of cells from the primary tumor, avoidance of detachment-induced cell death (anoikis), movement throughout the peritoneal cavity as individual cells and multi-cellular aggregates (MCAs), adhesion to and disruption of the mesothelial lining of the peritoneum, and submesothelial matrix anchoring and proliferation to generate widely disseminated metastases. This exceptional microenvironment is highly permissive for phenotypic plasticity, enabling mesenchymal-to-epithelial (MET) and epithelial-to-mesenchymal (EMT) transitions. In this review, we summarize current knowledge on EOC heterogeneity in an EMT context, outline major regulators of EMT in ovarian cancer, address controversies in EMT and EOC chemoresistance, and highlight computational modeling approaches toward understanding EMT/MET in EOC.

## 1. Introduction

Most epithelial carcinomas disseminate via the bloodstream or lymphatic system, utilizing a classical invasion-metastasis cascade mechanism which involves the local invasion of primary tumor epitheliocytes into the surrounding stroma and extracellular matrix (ECM), intravasation and transport through blood/lymph vessels, arrest at distant organ sites, extravasation into the organ parenchyma, and subsequent proliferation to form micro- and macro-metastases [1,2,3,4,5]. The triggering and ultimate success of these events depends on the epithelial-to-mesenchymal transition (EMT) and its key players, E-cadherin (epithelial, Ecad) [6] and N-cadherin (neural, Ncad) [7]–calcium–dependent transmembrane adhesion molecules which are responsible for maintaining cell-cell junctions between adjacent cells, thereby regulating the epithelial integrity and tissue architecture. During EMT, epithelial-type cancer cells undergo a set of molecular, morphological and functional changes with the loss of Ecad and gain of Ncad, which result in impaired epithelial cell-cell junctions and cell polarity, acquisition of a mesenchymal motile cell phenotype, and labile bonding with Ncad-expressing fibroblasts [8,9], endothelial cells and pericytes [10,11]. These changes facilitate cancer cell migration through stromal tissue, intravasation, and dissemination throughout the organism. The opposite process, designated mesenchymal-to-epithelial transition (MET), includes reverse cadherin switching (Ncad inhibition and Ecad re-expression) and often occurs at the secondary metastatic site, allowing for anchored and extravasating cancer cells regain epithelial features and proliferate into larger tumor nodules [2,3,12].

Epithelial ovarian cancer (EOC) is the deadliest gynecological malignancy, which stably ranks fifth highest among cancer deaths for women, and the American Cancer Society predicts that 14,080 women will die from ovarian cancer in 2017 [13,14]. The high mortality is primarily due to detection at late stages of the disease with vast intra-peritoneal dissemination [15] and to development of drug resistance after initial good response to treatment [16]. As opposed to other malignancies which progress through the above described canonical hematogenous invasion-metastasis cascade, EOC undertakes a distinct transcoelomic route of spread (through peritoneum-covered surfaces and organs of the abdominal and pelvic cavity), expanding via direct extension of cancer cells from the primary tumor into the intra-abdominal fluid-filled space, where they survive and travel as single cells and multi-cellular aggregates (MCAs) with the peritoneal fluid flow, subsequently adhering to peritoneal tissues, migrating into sub-mesothelial matrix and forming secondary lesions [17,18,19]. Recently, metastatic spreading of EOC via lymphatic [20] and blood [21,22] systems in vivo were reported. Nevertheless, the proposed new hematogenous models of EOC metastasis further highlight the involvement of the ovary in this process, as oophorectomy resulted in a complete abruption of peritoneal metastases and ascites development in mice [22]. These data suggest that, even in the case of hematogenous EOC cell circulation, metastatic EOC cells home to the ovary prior to further harnessing a predominantly intraperitoneal dissemination mechanism.

Hematogenously metastasizing solid tumors must first invade the tumor stroma and access the vasculature, necessitating an early EMT in order to adopt a motile, invasive phenotype. In contrast, the unique transcoelomic route of EOC dissemination generates an exceptional microenvironment quite distinct from most solid tumors, as cells are exfoliated directly into the peritoneal cavity. Thus, early events in metastatic dissemination do not require a mesenchymal phenotype. Alternatively, EOC exhibits phenotypic plasticity with regard to cadherin switching and exhibits significant cadherin heterogeneity during metastasis. In this review we focus on the peculiarities of the EMT/MET process in ovarian carcinoma, discuss tumor site-of-origin as a premise for EOC epithelial/mesenchymal heterogeneity, assess the potential clinical relevance of this plasticity, outline established and potential mediators of EMT/MET in EOC, and share our thoughts on possible future directions for EMT research.

## 2. EOC Cell of Origin: A Current Controversy

### 2.1. Ovarian Surface Epithelum Origin

**Figure 1:**
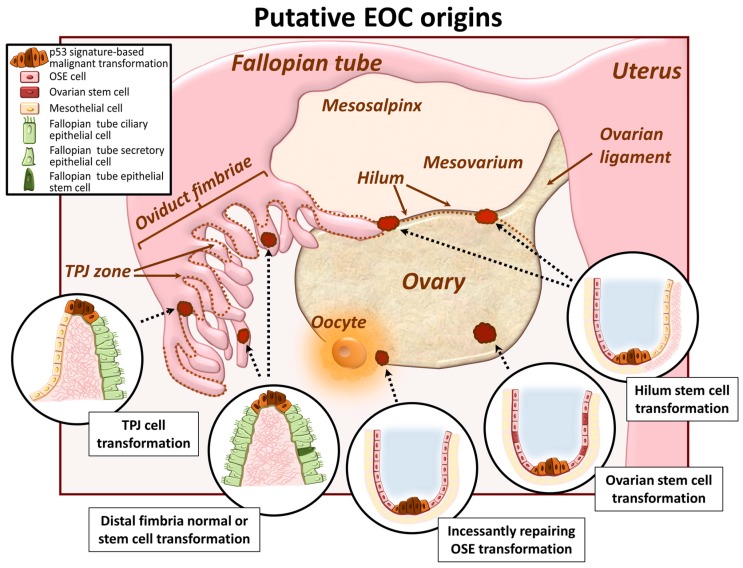
Putative epithelial ovarian cancer (EOC) origins. Represented is a schematic of the female reproductive system and the potential sites for ovarian cancer initiation. In the ovary, EOC may arise from the neoplastic conversion of the normal ovarian surface epithelium (OSE), as a result of frequent ovulatory rupture and post-ovulatory healing accompanied by transitions between mesenchymal and epithelial cell phenotypes; from malignantly transformed ovarian stem cells residing on the ovarian surface; or from the hilum, a translational zone between the ovary and mesothelium (and to some extent oviduct fimbriae), that contains cells with stem cell properties and propensity to tumorigenesis. In the fallopian tube, EOC may arise from the normal or stem-like cells in the distal fimbria actively participating in reproductive function-related fimbria injury; or from the tubal-peritoneal junction (TPJ), a region connecting mesothelium and oviductal fimbriae mucosa, often comprising translational metaplasia changes.

It was widely accepted for years that EOC arises from transformation of the normal ovarian surface epithelium (OSE), a mesodermally derived and hormone-dependent single cell layer that covers the ovary (Figure 1). The OSE regularly undergoes cycles of rupture and repair associated with ovulation and thereby flexibly shifts between mesenchymal and epithelial phenotypes in response to the need for migration and proliferation to regenerate the intact epithelial surface [23]. The normal OSE exhibits flat morphology with high expression of mesenchymal markers (Ncad, calretinin, mesothelin) and absence of epithelial markers (Ecad, EpCAM, EMA, OVGP1 and ciliary bodies) [24]. Conversion of OSE towards a cuboidal/columnar phenotype, indicative of tubal metaplasia of the OSE, is accompanied by acquisition of epithelial markers (Ecad, EpCAM, OVGP1, ciliary bodies) and suppression of mesenchymal markers (modestly downregulated Ncad and fully abrogated calretinin); alterations also characteristic of MET [24]. Among pathogenic factors suggested to initiate OSE metaplasia and malignant transformation is presence of inclusion cysts. The uneven ovarian surface contains invaginations and inclusion cysts that lead to overcrowding of OSE in these regions. Adaptation to a cuboid epithelial-like shape in these regions with accompanying metaplastic changes can then occur [25]. OSE cells trapped inside the inclusion cysts are more exposed to growth factors which may provide additional cues for neoplastic progression. OSE may also launch autocrine mechanisms through the release of hormones and cytokines [23]. Alternatively, OSE may undergo metaplasia to acquire Müllerian duct features with subsequent neoplastic progression to tumor formation [23].

### 2.2. Oviduct Fimbriae Epithelium Origin

In recent years, the epithelium of the distal fimbriae of the fallopian tube has been recognized as a potential site of origin of ovarian cancer (Figure 1). It is a pseudo-stratified columnar cell monolayer comprised of secretory and ciliated cells and exhibits both epithelial (high levels of Ecad, EpCAM, EMA, OVGP1) and mesenchymal markers (ubiquitous expression of Ncad and vimentin, and varying/absent expression of calretinin and mesothelin) [24,26]. Specifically, serous tubal intra-epithelial carcinoma (STIC), developed via acquisition of a p53 signature in a distal oviduct fimbriae tumor-initiating cell, is considered to be the site of tumor initiation, while tumors on the ovary and peritoneal surfaces are thought to be secondary lesions [27,28,29,30,31,32]. This hypothesis is supported by the fact that to date no premalignant lesions for ovarian carcinoma has yet been established. Furthermore, STICs can be found in patients without ovarian cancer, while ovarian and peritoneal malignancies are often associated with the presence of STIC [33]. Other aspects supporting this notion include the close physical proximity of the tube distal fimbria to the ovarian surface and the high frequency of identical p53 mutations in STICs and high grade serous adenocarcinomas [33]. Comprehensive analyses specifically focusing on accumulating evidence in support of both OSE and tubal fimbriae as sources of EOC initiation are published elsewhere [26,34].

### 2.3. Ovarian and/or Tubal Stem Cell Transformation

Recent findings propose stem cell transformation as a potential cause of ovarian cancer initiation (Figure 1) [35]. It has been postulated that ovarian carcinomas are heterogeneous tumors and contain a small number of cells with stem cell-like characteristics that express specific stem cell markers [35]. Retrieved from patient ascites, ovarian cancer cells positive for stem cell-related molecules (such as Oct4, Nestin, and Nanog) exhibit growth in an anchorage-independent manner in vitro and present serial metastatic tumors in vivo [35]. A large gene expression profiling study of normal OSE samples and patient-derived tissues from serous papillary adenocarcinoma patients revealed that a substantial pool of OSE cells are not fully differentiated (multipotent) and retain the capacity to serve as ovarian cancer initiating cells [36]. This side population of cells was identified and characterized from distinct genetically engineered mouse ovarian cancer cell lines, and showed the ability to efflux the DNA-binding dye Hoechst 33342, formed measurable tumors more rapidly and were less sensitive to the chemotherapeutic agent doxorubicin when compared with non-side population cells [18,37]. The detection of ovarian carcinoma stem cells with an ability to self-renew and high epithelial plasticity raises the interesting possibility that these cells have higher metastatic efficiency and may be responsible for the majority of metastasizing ovarian carcinoma cells [18]. Recently, a cancer-prone ovarian cancer stem cell niche was identified [38]. It constitutes the hilum region of the ovary, a translational (or junctional) area between the OSE, mesothelium and tubal (oviductal) epithelium [38]. The hilum region contains a large population of slowly cycling cells that express stem and progenitor cell markers and demonstrate a high propensity to malignant transformation [38]. Another possible “hot spot for carcinogenesis” was identified in a study that characterized the tubal-peritoneal junction (TPJ, a zone where peritoneal mesothelium and oviductal fimbria mucosa meet) with regions of translational metaplasia [39]. Finally, fallopian tube epithelium stem-like cells, lacking markers of ciliated or secretory differentiation, were isolated at the distal (fimbriated) end of the fallopian tube, at the site of frequent, reproductive function-associated fimbria injury, and may also serve as a starting point for EOC carcinogenesis [40]. An elegant report illustrating current knowledge about ovarian and tubal epithelium stem-like cells, their molecular properties and cell niches is published in [41].

## 3. Phenotypic Heterogeneity and Relevance of Intraperitoneally Residing Cells/MCAs

As described above, tumors classified as ovarian cancer can arise from histologically and molecularly distinct neoplastic progenitors: either from mesenchymal-type OSE that has undergone metaplastic MET changes; from the highly differentiated columnar fallopian tube epithelum which contains both epithelial (predominantly) and mesenchymal (moderately) traits; or from a variety of ovarian surface, oviductal fimbriae or junctional area-localized stem-like precursors which may diversely express epithelial, mesenchymal and stem cell markers. Given the unique intraperitoneal metastasis mode and the diversity of progenitors, it is not surprising that malignant ascites contains a heterogeneous pool of individual and clustered (as MCAs) free-floating metastatic units within the peritoneal cavity [17,42,43,44]. Furthermore, mechanisms that regulate metastatic MCA formation are currently unknown, however options include exfoliation of single cells from the primary tumor followed by association into aggregates; exfoliation of cell-cell adherent sheets from the primary tumor that subsequently condense into MCAs; or through proliferation of individual cells in suspension into aggregates.

The implications of the clinically observed epithelial, mesenchymal and intermediate cell phenotypes are a subject of continued active investigation. Recent studies show that ovarian cancer cell metastatic properties, aggregation dynamics, MCA surface morphology, inner ultrastructure and behavior differ among epithelial, intermediate and mesenchymal phenotypes and are regulated by E- and Ncad expression both in their free-floating state [43] and at the secondary metastatic site [45]. It has been reported that mesenchymal-type EOC cells tend to aggregate into very compact solid spheroids, whereas epithelial-type EOC form loose and easily disaggregated MCAs [43], and these properties are modulated by shifts in E- and N-cadherin expression. These findings are interesting in the content of MCA survival and chemosensitivity, as such differences in aggregate cohesivity may drive distinct responses of epithelial and mesenchymal MCAs to hypoxic conditions, glucose starvation, and drug penetration to cells in the MCA core.

### 3.1. Mesenchymal Phenotype

**Figure 2:**
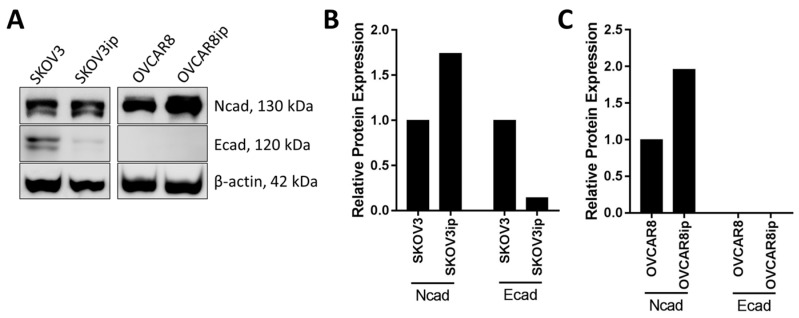
Cadherin expression alterations in human EOC cell lines and their highly metastatic derivatives. (**A**) Ecad and Ncad expression in parental SKOV3 and OVCAR8 cells and their matching highly metastatic derivatives, SKOV3ip and OVCAR8ip (isolated from murine intraperitoneal metastases), was assessed by Western blot analysis with the rabbit monoclonal anti-Ecad (Abcam, 1:1000 dilution) or mouse anti-Ncad (Life Technologies, 1:1000 dilution) primary antibody, respectively, followed by peroxidase-conjugated anti-rabbit or anti-mouse secondary antibody (Sigma-Aldrich, 1:4000 dilution) and enhanced chemiluminescence detection by ImageQuant LAS4000 biomolecular imager. (**B**,**C**) The densitometric analysis of immunoblot bands represented in Figure 2A was conducted in ImageJ, and protein expression (band relative intensity) was normalized against β-actin loading control.

A recent study [45] delineated that within a mixed population of EOC cells and MCAs, only mesenchymal cells and mesenchymal cell-containing aggregates exhibit the ability to invade through 3-dimensional (3D) collagen-rich matrices. Moreover, this process relies on competent Ncad cell-cell junctions and can be successfully blocked by N-cadherin antagonists. Overexpression of Ncad also stimulated mesothelial cell clearance by cancer MCAs [45]. These findings may provide a partial explanation for differential metastatic efficiency among various ovarian cancer cell lines in murine models. Epithelial type ovarian cancer cells, such as OvCa433, do not readily form intraperitoneal tumor xenografts [46], and in vivo growth of this cell line is limited to subcutaneously injected regions [47]. Meanwhile, mesenchymal-type (Ncad+) cell lines easily create large numerous widely-disseminated metastatic lesions in vivo, often accompanied by cancerous cachexia and ascites in mice [48,49,50]. An immunoblot analysis of two metastatically successful EOC cell lines (SKOV3 and OVCAR8) and their highly metastatic in vivo-selected intraperitoneal (ip) derivatives (SKOV3ip and OVCAR8ip), revealed elevated Ncad in the absence of Ecad in aggressive ip sublines (Figure 2). This is similar to data obtained from analysis of primary tumors and patient-matched metastatic lesions, wherein enhanced Ncad expression is observed in metastases relative to the corresponding primary tumor [17]. Together these data suggest that intraperitoneal delivery of Ncad blocking molecules could be beneficial for suppressing development of metastatic lesions. Moreover, given that EMT is often a reversible process, selective targeting of EMT mediators to induce a MET program in mesenchymal-type EOC cells [12] may become potentially advantageous against peritoneal lesion formation.

### 3.2. Epithelial Phenotype

Epithelial phenotype cells may also promote EOC progression. Existing literature suggests that enhanced Ecad expression in early stages of EOC progression is necessary to avoid detachment-induced apoptosis and for resistance to radiation and chemotherapy [51,52,53,54,55]. In agreement with these observations are studies reporting enhanced chemoresistance in EOC cells of epithelial phenotype as compared to mesenchymal phenotype (see Section 5 for further discussion) [56,57]. One the other hand, loss of Ecad is canonically associated with the disruption of adherens junctions, and shedding of the Ecad extracellular domain induced by different bioactive molecules (lysophosphatidic acid, epidermal growth factor, etc.) leads to increased detachment of EOC cells from the tumor surface [23,58,59,60,61]. Besides the loss of cell-cell junctions and exfoliation/dissemination of more tumor cells into the peritoneal cavity, the shed Ecad ectodomain retains functional significance and was documented to further disrupt existing cell junctions between EOC cells [62], stimulate cancer cell invasion by triggering signaling pathways [63], and upregulate matrix metalloproteinase (MMP) MMP-2, MMP-9 and membrane type 1 (MT1-MMP) expression [64]. Meanwhile, the freed cytoplasmic domain of Ecad may also potentiate cancer progression via Wnt signaling, as the release of junctional Ecad and β-catenin leads to nuclear accumulation of soluble β-catenin and enhanced transcriptional activity [65]. In some cases (as reported for esophageal squamous cell carcinoma), nuclear translocation of the cytoplasmic Ecad fragment can alone promote transcriptional regulation, independently of β-catenin recruitment [66]. More on the role of Ecad and the functional significance of its cleaved domains is reviewed in [67,68].

### 3.3. Intermediate (Hybrid) Phenotype

**Figure 3:**
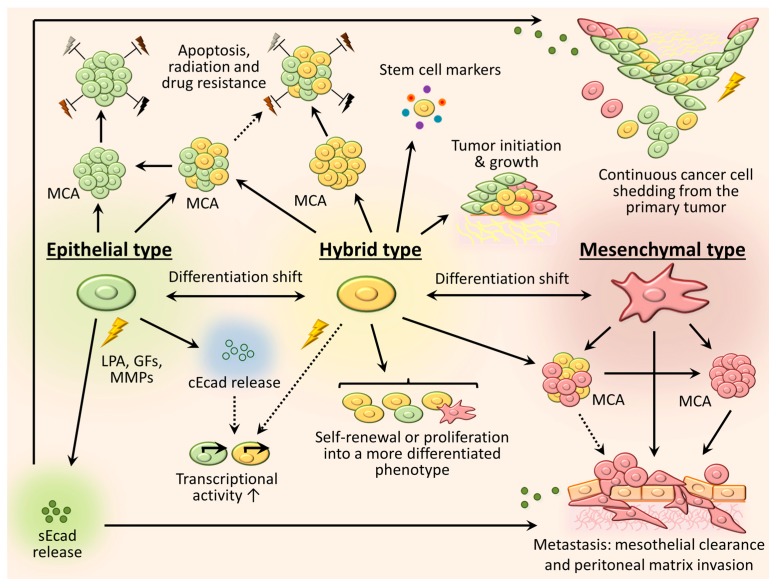
Phenotypic plasticity of EOC cells in the peritoneal cavity. Triggered by the soluble factors abundant in intraperitoneal fluid such as the bioactive lipid lysophosphatidic acid (LPA), growth factors (GFs), and matrix metalloproteinases (MMPs) (all designated as yellow lightening), ovarian cancer cells exist as a heterogeneous mixture of free-floating epithelial, mesenchymal and intermediate (hybrid) cells inside the peritoneal cavity. These cells can dynamically shift between phenotypes along the EMT spectrum adjusting to microenvironmental cues. Multicellular aggregation (MCA) of epithelial-type cells (green) is reported to play a pro-survival role through developing resistance to cell death, radiation and chemotherapeutics (designated grey, brown and black lightening). Bioactive molecules promote cleavage of the Ecad ectodomain to generate a soluble Ecad fragment (sEcad, green filled dots). The sEcad fragment may promote further disruption of cell-cell junctions between the epithelial and hybrid cells and enhance cell shedding from the primary tumor surface. The released Ecad cytoplasmic domain (cEcad, green open dots) may stimulate cell transcriptional activity. Mesenchymal-type cells (pink) readily form MCAs and are predominantly involved in metastasis-associated behaviors, exhibiting mesothelial cell (orange cuboid) clearance activity and peritoneal matrix invasion. Additionally, cell invasion may be amplified by stimuli from sEcad. The hybrid cells (yellow) retain stemness properties: expression of stem cell markers (colored dots), ability for self-renewal or proliferation into a more differentiated phenotype, tumor initiation (tumor-initiating cell designated red-circled) and growth. Hybrid cells may also form mixed MCAs with either epithelial or mesenchymal cells, undergo differentiation consistent with the phenotype of the adjacent cells in co-culture and share their subsequent fate. Black arrows represent interactions reported in ovarian carcinomas; dashed arrows designate patterns that were observed in other cancer types and may potentially be applicable towards ovarian cancer, but require further validation.

It is currently well recognized for many malignancies that cancer cells do not transiently exhibit hybrid epithelial/mesenchymal (E/M) properties, but rather stably maintain a certain level of intermediate (partial EMT, semi-EMT) phenotype [69,70,71,72], which provides benefits over purely epithelial or mesenchymal morphological states. As widely reported in literature, the hybrid E/M phenotype allows cells exist dynamically, adjusting their differentiation and functionality in response to the environmental milieu [69,73,74,75,76], garner resistance to cell death, radiation and chemotherapeutic agents [69,77,78,79]. Moreover, multiple studies indicate that in contrast to cells in a finite epithelial or mesenchymal state, hybrid E/M cells more readily display stemness properties, such as multipotency and self-perpetuation [80,81], (re)-expression of stem cell markers [76,80,81], sphere formation [80,82] and tumor-initiating potential [81,82]. For ovarian cancer in particular, a subset of in vivo tumorigenic cells have been detected in a hybrid E/M state [81], which simultaneously express epithelial, mesenchymal and cancer stem cell markers, and retain the capacity for self-renewal as well as generation of more differentiated progenies. The authors demonstrated that the differentiation fate of the hybrid E/M ovarian cancer cells is associated with EMT pathways and may be regulated by environmental stimuli or the state of adjacent (epithelial or mesenchymal) cells [81]. Similarly, dual cadherin-expressing (intermediate) EOC cell lines OVCAR3 and OvCa432 proliferate to create a heterogeneous mixture containing hybrid Ecad+/Ncad+, predominantly Ecad+ and predominantly Ncad+ progenies within the same subclone, as confirmed by immunostain analysis [43]. Furthermore, the newly generated hybrid Ecad+/Ncad+ subclones exhibit higher proliferation indices in comparison with their parental, purely epithelial or purely mesenchymal EOC cells [43]. Additionally, substratum-free EOC cell aggregation relies on homotypic Ecad:Ecad or Ncad:Ncad interactions; as a consequence, EOC cells in co-culture exhibit the propensity to sort into purely epithelial Ecad-expressing and purely mesenchymal Ncad+ MCAs, whereas hybrid cells presenting both cadherins can form heterogeneous MCAs with all cell phenotypes [43]. This may serve an additional tumor-supporting role through recruitment of these stem-like cells adjacent to fully differentiated cells (e.g., to the metastatic niche together with mesenchymal-type cells or as an apoptosis-resistant component of epithelial-type aggregates). Our current understanding of EOC cell/MCA phenotypic heterogeneity, plasticity and the significance for ovarian cancer progression is summarized in Figure 3.

## 4. Factors Contributing to Dynamic EMT Shifts in Ovarian Cancer Cells

### 4.1. Components of Ascitic Fluid

**Figure 4:**
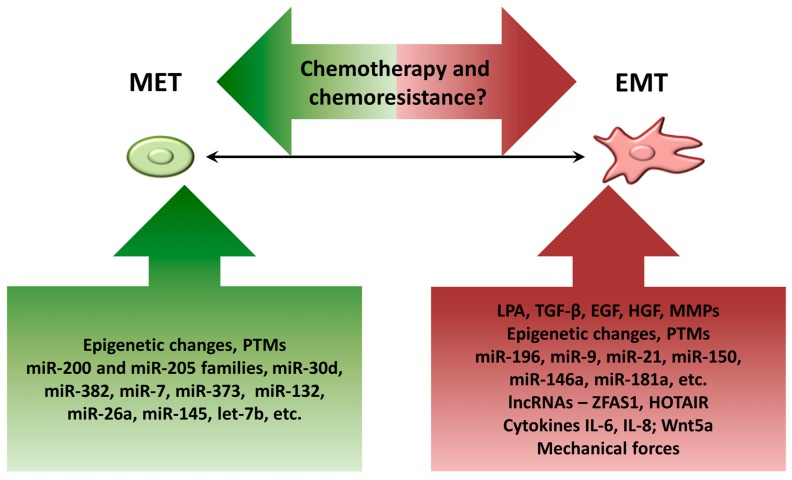
Overview of EMT regulators in ovarian cancer. The diagram summarizes major factors involved in MET (green box) and EMT (red box) programs during EOC progression. The interplay between EMT shifts and ovarian cancer chemotherapy/chemoresistance currently remains controversial.

While MET processes accompanying ovarian carcinoma initiation usually occur due to genetic alterations (neoplastic metamorphosis built on a p53 signature background as a consequence of incessantly damaged OSE or distal oviduct fimbriae, or vulnerabilities of the translational metaplasia regions in the areas of junction between two distinct epithelia), the subsequent shifts along the MET/EMT spectrum in intraperitoneally-residing cells are due not to genetic mutations, but rather occur as a response to a variety of external cues arising from the ascitic microenvironment (Figure 4).

One of the most well-characterized factors implicated in ovarian carcinogenesis and metastasis is lysophosphatidic acid (LPA), a bioactive lipid molecule which is notably abundant in ascites and plasma of patients with ovarian cancer and activates a subfamily of G-protein coupled cell surface receptors, eliciting a variety of cellular responses such as aberrant proliferation, adhesion, migration, invasion and anoikis-resistance [83,84,85,86,87,88]. It was shown that LPA is constitutively produced by the mesothelial cells of the peritoneum, increasing EOC cell adhesive, migratory and invasive properties [89]. In the content of EMT, LPA was shown to induce shedding of the 80 kDa extracellular domain of Ecad from EOC cells in a urokinase plasminogen activator (uPA)-dependent manner, disrupting cell-cell junctions and promoting a cellular mesenchymal phenotype with enhanced motility and invasion [63]. Notably, in this work the authors showed that the cleaved soluble Ecad itself also stimulates EOC cell invasion [63]. Additionally, LPA upregulates expression of matrix metalloproteinase-9 (MMP-9) and triggers MMP-9-catalyzed Ecad ectodomain shedding in a concentration- and time-dependent manner, disrupting junctional integrity in ovarian cancer cells and contributing to EMT [60]. Notably, blocking LPA receptors successfully suppressed MMP-9 expression levels and restored EOC cell-cell adhesion [60], suggesting a possible approach for therapeutic intervention. LPA is also involved in reorganization of the actin cytoskeleton, causing rearrangement of stress fibers and cell adhesion together with enhancement of MMP-2 enzymatic activity [90]. MMP-2 activation is enhanced in LPA-treated cells and results in enhanced MMP-dependent migratory and invasive behavior of EOC cells [91]. Following the disruption of cell-cell junctions, LPA also initiates loss of junctional β-catenin, promotes its nuclear translocation and reinforces the EMT program in ovarian cancer cells via activation of Wnt/β-catenin signaling nexus [92]. Recently, LPA was elucidated to stimulate the epithelial-to-mesenchymal switch in ovarian cancer cells via downregulation of SIRT1, an established inactivator of ZEB1 and suppressor of EMT [93]. Additionally, LPA intensifies EMT in ovarian cancer through upregulation of Slug/Snail2 EMT markers via Gαi2, Src, and HIF1α signaling pathway [94].

In addition to LPA, other growth factors, such as epidermal growth factor (EGF), hepatocyte growth factor (HGF), transforming growth factor-β (TGF-β), present in ovarian neoplastic microenvironment, are thought to influence EMT by inducing cleavage of Ecad fragments to impair cell-cell cohesion and via enhancing multiple complex signaling networks and transcriptional activity. These studies are described elsewhere [95,96,97,98].

Several MMP family members are known to be involved in EMT processes for different types of epithelial cancers, including ovarian carcinomas. Due to their proteolytic activity, multiple MMPs reportedly cause cleavage of the Ecad ectodomain and reorganization of zonula occludens (repression of tight junction protein ZO-1), hence, abrogating cell-cell adhesion, augmenting β-catenin nuclear translocation and activation of Wnt signaling, boosting acquisition of mesenchymal traits by cancer cells and enhancing cell invasiveness both in vitro and in vivo [60,91,99,100,101,102,103]. The later process is also reinforced due to the well-known role of MMPs in proteolysis and remodeling of diverse components of extracellular matrices [104,105,106]. Notably, MMP expression and activity may in turn be regulated by EMT-associated factors, such as Snail [107] or β-catenin [108,109,110]. More on the diverse effects of MMPs on cancer progression and metastasis, their targets, regulation mechanisms and potential therapeutic implications is discussed in [111,112,113].

Ovarian cancer cells are also subjected to cytokines and immune cells present in EOC-associated malignant ascites, that may potentially contribute to EMT shifts. In particular, interleukins (IL)-1β, IL-6, IL-8 and IL-10 are substantially abundant in ascitic effusions from EOC patients ([114,115,116] and reviewed in [117]). Notably, the literature reports a pro-EMT role for IL-6 and IL-8 in ovarian cancer. Attenuated Ecad expression, upregulation of β-catenin and enhanced SKOV3 and OVCAR3 cell migration in response to IL8 has been demonstrated [118]. Similarly, exposure of EOC and other gynecologic cancer cells to IL-6 treatment mitigates epithelial marker expression and elevates mesenchymal markers expression, increases MMP-2 and MMP-9 activity and avidly enhances migratory and invasive properties [119]. Additionally, IL-6 is known to be involved in EMT shifts, self-renewal, inducing stemness properties in cancer cells and drug resistance (outlined in [120]). Interestingly, production of both IL-6 and IL-8 is reinforced by LPA through transcriptional activation of IL gene promoters and upregulation of LPA receptors [121]. Evidence for chemokine ligand 5 (CCL-5) and chemokine receptors (CCR)-1/3/5 [122], CCL-19/21 and CCR-7 [123] involvement in EMT events and ovarian carcinoma metastasis have also been reported.

Other bioactive molecules may also participate in the EMT/MET program in EOC. For example, the mucin CA125/MUC16 may be associated with EMT in ovarian cancer due to its binding to Ecad and β-catenin complexes, as its downregulation results in epithelial-mesenchymal switch (as evidenced by loss of Ecad and cytokeratin-18 and gain of Ncad and vimentin), re-activation of EGFR signaling and increases in MMP-2 and MMP-9 expression and functional activity [124]. Contrary to these results are data [125] demonstrating increased EOC tumor growth, colony formation, cell motility, invasive and metastatic properties upon overexpression of MUC16, accompanied by loss of Ecad and enhancement of Ncad and vimentin expression. Non-canonical Wnt5a signaling is another potential EMT-regulator and has recently been the subject of a comprehensive review [126]. Wnt5a protein is rich in ovarian cancer patients’ malignant ascites [126] and its expression is shown to correlate with vascular mimicry and metastatic success [127]. Therefore, additional research to elucidate the role of Wnt5a in EOC progression and metastasis is warranted.

### 4.2. Epigenetic Changes

EMT-related epigenetic changes have been described for many cancers including EOC [128]. Silencing of CDH1 (Ecad) by promoter hypermethylation takes place in breast, prostate, gastric and urinary bladder cancers [129,130,131]. Zinc finger E-box-binding homeobox 1 (ZEB1), a known transcriptional repressor of Ecad, can also work epigenetically through recruitment of a DNA methyltransferase 1 (DNMT1) to the CDH1 promoter [132]. Among the most common histone modifications involved in EMT are histone demethylation at the SNAI1 promoter (activate transcription of SNAI1), di- or trimethylation at the TWIST promoter (TWIST activation), and histone mono-, di- and trimethylation at the CDH1 promoter by multiple methyltransferases (CDH1 transcription suppression) [128,133,134,135,136]. Histone acetylation (recruitment of histone acetyl transferases, HATs, and transcription activation) and deacetylation (recruitment of histone deacetylases, HDACs, and transcription inactivation) are mechanisms often employed for regulation of Ecad expression. SNAI1 and ZEB1 use HDACs for Ecad silencing [137,138]. TWIST recruits a multi-protein complex capable of deacetylation and nucleosome remodeling to the Ecad promoter, thus suppressing Ecad transcription [139]. On the contrary, reactivation of Ecad expression can result from deacetylation of its transcriptional repressor SNAI2 (SLUG) [140]. A comparative analysis of epigenetic events in breast and ovarian cancer related to tumorigenesis [141] identified genetic, microenvironmental, stromal, and epigenetic changes common between breast and ovarian cancer cells, as well as the clinical relevance of these changes. Some of the most striking commonalities included epigenetic alterations in H3K27me3, H3K9me2, H3K9me3, H4K20me3, and H3K4me suggesting shared features of pathogenesis in EOC and breast cancer and pointing out novel directions for managing EOC progression. Epigenetic profiling of ovarian cancer cells following TGF-β treatment discovered significant hypermethylation changes in the loci associated with EMT and cellular movement amongst others [142]. Moreover, the authors showed that TGF-β also stimulated expression and activity of DNMTs, while treatment with DNMT inhibitor SGI-110 abrogated TGF-β-mediated EMT [142]. Epigenetic silencing of secreted frizzled-related protein 5 (SFRP5), important in Wnt signaling regulation, activates the later and thus promotes EOC progression and drug resistance through TWIST-mediated EMT and AKT2 signaling [143]. IQGAP2 was found to be significantly hypermethylated in EOC [144] showing an inverse correlation between IQGAP2 DNA methylation and mRNA expression as IQGAP2 expression was downregulated in EOC. The subsequent survival analysis revealed that decreased IQGAP2 was associated with the low progression-free survival of EOC patients. Moreover, IQGAP2 was shown to suppress Wnt-induced β-catenin nuclear translocation and transcriptional activity, therefore inhibiting EMT, cell invasion and migration. Thus, IQGAP2 was identified as a novel EOC tumor suppressor via repression of invasion through Wnt/β-catenin signaling and was suggested as a new potential therapeutic strategy for EOC treatment. Numerous DNA methylation aberrations have been recognized in EOC. The examples provided above and summarized in Table 1 highlight the potential clinical implications as novel biomarkers for EOC diagnosis and disease progression. More details on candidate genes for epigenetic therapy of EOC can be found in [145,146,147].

### 4.3. Posttranslational Modifications (PTMs)

The most commonly present PTMs mediating EMT include phosphorylation and glycosylation. Phosphorylation of SNAI1 motifs by different protein kinases (such as GSK-3β and protein kinase D1) subsequently initiates its ubiquitination and degradation, thereby restoring expression of Ecad [148,149,150]. Inhibition of these protein kinases leads to Ecad repression and EMT. By contrast, glycosylation of SNAIL stabilizes it by preventing its phosphorylation and subsequent degradation, thus supporting EMT activation [151]. Another PTM, SUMOylation (binding of a small ubiquitin-like modifier, SUMO, to the target transcription factor) acts in a suppressive manner and is important for stabilizing and deactivating transcription factors engaged in EMT. In particular, in breast cancer the transcription factor Forkhead box protein M1 (FoxM1) is known to induce EMT via SNAI2 (SLUG) promoter stimulation [152], while in pancreatic cancer, FoxM1 upregulates ZEB1, ZEB2, SNAI2 and vimentin [153]. Yet another transcription factor Smad-interacting protein 1 (SIP1) acts as a downregulator of Ecad [154]. Impaired SUMOylation contributes to constant activation of these factors and, thus, enhances cadherin switching and EMT. One of the important promoters of EOC is the Hippo pathway signaling, which has been shown to affect several key signaling molecules via various types of PTMs [155,156]. In particular, the malfunction of critical Hippo signaling modules such as YAP/TAZ, MAT1/2 and LATS1/2 due to deregulated PTMs has been linked to different types of cancer including EOC. The current knowledge of PTMs with respect to the Hippo signaling pathway and possible therapeutic interventions targeting PTMs and Hippo signaling have been recently reviewed in [155]. Additional information on mediation of EMT at the post-translational level and an overview of various therapeutic approaches currently being investigated to undermine EMT can be found in [128].

### 4.4. MicroRNAs

Multiple microRNAs have been reported to serve as EMT promoters or inhibitors [157,158]. Members of the miR-205 and miR-200 family suppress EMT by binding the mRNAs encoding ZEB1 and ZEB2 [159,160]. Interestingly, both miR-205 and miR-200 family members negatively correlate with the expression of another EMT driver TWIST1, and are themselves transcriptionally silenced by TWIST1, which directly binds miR-205/miR-200 promoters and further intensifies EMT, complementing its well-known Ecad-suppressive role [161]. MiR-132/212 tandem miRNAs inhibit TGF-β-associated EMT by blocking the SOX4 gene in prostate cancer [162] and alleviate EMT and invasion of cervical cancer via SMAD2 downregulation [163]. Mir-132 directly silences ZEB2, thus attenuating EMT, invasion and metastasis in colorectal [164] and lung [165] cancers, and reduces EMT and migratory/invasive capacity of human non-small cell lung carcinoma (NSCLC) through the EMT-related TGF-β1/Smad2 pathway [166]. MiR-150 triggers EMT and metastatic behavior in NSCLC in vitro and in vivo through downregulation of FOXO4 [167]. In contrast, in esophageal squamous cell carcinoma, miR-150 induces MET-like changes and blocks murine xenograft tumor growth by targeting ZEB1 [168]. MiR-9 stimulates EMT via immediate repression of Ecad-encoding mRNA [169]. In lung cancer-initiating cells, miR-145 downregulates stem-like properties and EMT via blocking Oct4 [170]; alternatively, in NSCLC, miR-145 and -203 are involved in TGF-β-related EMT inhibition through targeting SMAD3 [171]. Among other known pro-metastatic (pro-EMT) microRNAs are miR-27 [172,173], miR-29a [174,175,176], miR-103/107 [177,178], miR-221/222 [179,180,181] and miR-661 [182]; alternatively, miR-26a [183,184], miR-26a-5p [185,186], miR-30a [187,188,189], miR-30a-5p [190], miR-134 [191], miR-194 [192,193,194], miR-192 and -215 [194], and miR-204 [195,196,197,198] exhibit anti-EMT activity.

A large number of microRNAs are implicated in ovarian carcinogenesis, tumor progression and in EMT in particular. A comparative study investigating expression of the miR-200 family, ZEB1 and ZEB2 transcriptional repressors in normal OSE vs. 15 EOC cell lines and 70 ovarian carcinoma tissues revealed that malignant transformation is associated with acquisition of more epithelial traits, such as upregulation of miR-200 family members and attenuation of ZEB1/2 [199], supporting the occurrence of MET, not EMT, during early stages of ovarian cancer progression. The miR-196 family, located in the Hox gene cluster, regulates (usually by inhibition) the HOX genes, in particular HOXA7, which is responsible for controlling the differentiation status in ovarian epithelium. HOXA7 overexpression is associated with the initiation of MET in ovarian epithelium and generation of low-grade Ecad-positive ovarian tumors [200]. Mir-9 levels are often elevated in ovarian cancer tissues in comparison with normal control tissues and are associated with EMT via targeting of Ecad by miR-9 and consequent enhancement of Ncad and vimentin expression [201]. Transcriptional regulator Snail is the direct target of miR-30d, which inhibits TGF-β1- induced EMT in ovarian cancer cells [202]. MiR-424 can suppress cell invasion and EMT via downregulation of DCLK1 in ovarian clear cell carcinoma, a subtype of EOC, associated with drug resistance and low survival rate [203]. MiR-382 serves as an ovarian cancer suppressor due to regulating EMT by targeting ROR1 and negatively impacting EOC cell migration/invasion [204]. A recently discovered miR-506 is one of the crucial down-regulators of EMT and metastasis in EOC through both immediate repression of transcriptional repressor SNAI2 (thus, restoring Ecad expression) and negative regulation of Ncad and vimentin, and is associated with a better survival prognosis [205]. Another EOC EMT- and metastasis-suppressor is miR-7, which acts through EGFR and AKT/ERK1/2 pathway inactivation [206]. More microRNA aberrations observed in EOC are concisely summarized in a review [207].

The concept of microRNA detection in physiological fluids in order to establish new predictive and diagnostic markers is gaining in popularity. A recent analysis of serum-circulating exosomal microRNAs revealed a broad increase in miR-373 and miR-200a in patients with ovarian serous adenocarcinoma across all stages (I-IV), while miR-200b and miR200c were more significantly elevated in later stages (III-IV) and correlated with worse survival outcome, suggesting that these microRNAs may differentially modulate EMT/MET shifts during certain EOC progression steps [208]. Another study [209] evaluating 2222 total microRNAs from ovarian cancer patient serum samples identified the most stably and markedly downregulated microRNAs as miR-132, miR-26a, and miR-145 (which are known to act as EMT-repressors in other tissue types, as discussed above), as well as let-7b, a microRNA that was recently shown to play multiple anti-tumor roles, including anti-EMT (through attenuation of p-AKT, Twist and β-catenin) and pro-apoptosis in malignant mesothelioma cells [210]. A 1,170 patient-based meta-analysis of global transcriptome data delineated let-7b as a stratification factor for molecular and clinical classification, and a predictor of poor survival outcome in high grade serous ovarian carcinoma [211]. Urinary miR-30a-5p was recently reported to be exclusively elevated in ovarian serous adenocarcinoma patients, both at early and metastatic stages of the malignancy and derived specifically from EOC tissue [212]. In contrast, elevation of miR-30a-5p in patients with benign gynecological diseases, gastrointestinal tumors and in the healthy control group was not detected. Moreover, targeted inhibition of miR-30a-5p considerably mitigated ovarian cancer pro-metastatic behavior in vitro [212]. In addition to EMT mediation, the majority of involved microRNAs play multiple roles in ovarian cancer initiation, progression, metastasis, stemness, microenvironmental and chemotherapeutic responses. In a paired analysis, miR-21, miR-150, and miR-146a were shown to be significantly overexpressed in EOC omental metastatic lesions in comparison with their matching primary tumor samples from the same patients [213]; moreover, miR-150 and -146a were also shown to regulate the size of EOC multicellular spheroids, cell survival and chemoresistance to cisplatin [213]. Amongst others, miR-181a is shown to facilitate TGF-β-mediated EMT through downregulation of SMAD7, contributing to cell survival, metastasis-associated behavior and chemoresistance in high grade serous ovarian carcinomas [214]. Further elucidation of signaling pathways and molecular targets of clinically dysregulated microRNAs in EOC is of profound importance, warranting discovery of new predictive markers, diagnostic tools and targeted therapeutic strategies. A comprehensive analysis (2014) based on evaluation of almost 100 research publications provides a structured summary of potentially relevant prognostic, diagnostic and therapeutic microRNAs for ovarian cancer [215]. Yet, another thorough review summarizes the most recent advances in clinical applications (including current clinical trials) of microRNAs for ovarian cancer precision medicine [216].

### 4.5. Long Noncoding RNAs (lncRNAs)

LncRNAs are a large class of RNAs transcripts having a length of more than 200 nucleotides which do not encode proteins. LncRNAs have gained widespread attention in recent years due to the vast implications in cancer biology, contributing to essential cellular functions including invasion, proliferation, differentiation, apoptosis, cell cycle progression and metastasis [217,218,219,220]. Although emerging as a new class of regulators in cancer progression, the role of lncRNAs in EOC and the relationship to EMT/MET in EOC has just begun to be explored.

A comprehensive study using clinical data from 544 ovarian cancer patients from The Cancer Genome Atlas (TCGA) examined lncRNA expression profiles [221], identifying an eight-lncRNA signature (RP4-799P18.3; PTPRD-AS1; RP11-57P19.1; RP11-307C12.11; RP11-254I22.1; RP11-80H5.7; RP1-223E5.4; GACAT3) permitting classification of patients into two groups characterized as high-risk + poor outcome and low-risk + significantly improved outcome. In particular, a superior prognosis performance in BRCA1/2-mutated and BRCA1/2 wild-type tumors was achieved by associating predictions with BRCA1 and BRCA2 mutations. It was shown that the eight-lncRNA signature may serve as a metric to predict chemotherapy response in patients and identify resistance to platinum treatment suggesting other more efficient therapies. These findings support that lncRNAs can be used as diagnostic or prognostic biomarkers in patients with EOC.

Upregulation of the lncRNA ZFAS1 was recently reported in EOC and negatively correlated with overall survival of patients with ovarian carcinomas [218]. It was established that overexpression of ZFAS1 increased proliferation, migration and chemoresistance in EOC cells. miR-150-5p was identified as a potential target of ZFAS1, suppressing the transcription factor Sp1. Meanwhile, inhibition of miR-150-5p partially restored proliferation and migration resulting from depletion of ZFAS1. Thus, the ZFAS1/miR-150-5p/Sp1 pathway was shown to be critical in inducing migration, differentiation and chemoresistance in EOC.

In another study [222], lncRNA HOX transcript antisense RNA (HOTAIR) expression in EOC tissues was evaluated and its correlation with clinico-pathological factors was established. Suppression of HOTAIR in three highly metastatic EOC cell lines (SKOV3.ip1, HO8910-PM, and HEY-A8) significantly reduced cell migration and invasion. Furthermore, the pro-metastatic effects were partially mediated by MMPs along with EMT-related genes. Specifically, siRNA-mediated silencing of HOTAIR increased expression of Ecad and decreased expression of vimentin and Snail.

TGF-β signaling has been shown to serve as a major promoting factor of EMT, facilitating EOC and breast cancer metastasis. Although the relationship between lncRNA and TGF-β in EOC is not known, the lncRNA profile in mouse mammary epithelial NMuMG cells upon TGF-β-mediated induction of EMT has been reported [223], identifying a subset of lncRNAs dysregulated upon TGF-β induced EMT with lncRNA-HIT mediating this process by targeting Ecad. These findings reveal a pivotal role that lncRNAs may play in EMT in breast cancer progression and warrant further studies examining relation between lncRNA profile and TGF-β-induced EMT in EOC. Collectively, these studies suggest a direct or indirect relationship between lncRNA and regulation of EOC invasion and metastasis, as well as new mechanisms involved in EOC EMT, which can potentially result in novel markers and therapeutic targets for epithelial ovarian cancer.

### 4.6. Biomechanical Forces

The unique transcoelomic route of ovarian cancer metastasis with excessive dissemination within the abdominal cavity leads to obstruction of lymphatic tissues, enriched in peritoneal walls, and early malfunction of the peritoneal lymphatic drainage system [224,225,226]. Excessive neovascularization of the primary tumor and peritoneal lining [227,228,229,230,231], secretion of factors increasing vascular permeability [232,233,234,235,236] and as a consequence, high protein concentration [237], motivate intraperitoneal transudate aspiration. A disrupted balance between fluid production and fluid clearance generates large volumes of ascites, followed by a dramatic increase in intra-peritoneal pressure (IPP) [238] which subsequently alters intraperitoneal mechanobiology. Meanwhile, biomechanical cues, driven by fluid build-up, ascitic currents, stretching of the peritoneal tissues and organ dislocation currently remain relatively unexplored. One published study has shown that laminar fluid flow drives a more aggressive phenotype in 3-dimensional ovarian micro-nodules through induction of EMT, partially via post-translational upregulation of EGFR, as well as significant downregulation of Ecad and CDC2 expression [239]. These results are in conformity with a study reporting triggering of EMT in laryngeal squamous cell carcinoma by fluid shear stress through integrin-ILK/PI3K-AKT-Snail signaling pathways [240]. Exposure to shear stress caused repression of Ecad with a simultaneous increase in Ncad expression and translocation of β-catenin into the nucleus, alteration of cell morphology to an elongated shape with invadopodia enrichment, and stimulated cell migration; furthermore, these alterations were reversible upon removal of mechanical stress [240]. Conversely, another study addressing the effects of fluid sheer stress on EMT and cancer stem cell (CSC) properties in breast cancer cells documented CSC-like signature promotion with no changes in EMT markers [241]. Peritoneal fluid dwell was shown to drive EMT and hyperplasia of mesothelial cells in an in vitro reconstructed peritoneal cavity model [242]. A transcriptome-wide analysis of microRNA profiles in breast cancer cells and cancer-associated fibroblasts in response to compressive strain revealed alterations in expression levels of microRNAs associated with EMT, migration, invasion, angiogenesis and apoptosis [243]. In 3D breast cancer aggregates, elevated interstitial fluid pressure guided EMT shifting and collective cell invasion through Snail, vimentin and Ecad gene expression alterations [244]. In normal and hypertrophic scar fibroblasts, mechanical compression altered expression of TGF-β signaling target genes SMAD2 and SMAD3 and further upregulated expression of MMP-2 and MMP-9 [245]. Potential alterations in ovarian cancer progression mediated by ascites-induced changes in peritoneal mechanobiology have yet to be uncovered. One recent study has revealed significant enhancement of SNAI1 gene expression in epithelial-type EOC cells and MCAs upon continuous patho-physiologically relevant compression (~22 mmHg), applied to mimic ascites-driven elevations in IPP as observed in the clinic [246]. Additionally, elevated mRNA and protein expression levels of Ecad and Ncad in EOC epithelial- and mesenchymal-type MCAs, respectively, heightened the distinction between these morphological phenotypes [246].

## 5. Controversy on EMT and Chemoresistance in EOC

Progress made in elucidation of EMT as a continuously evolving process rather than two distinct end stages has heightened controversies related to the divergent behavior of epithelial and mesenchymal cancer cells during metastatic spreading and their response to therapeutic interventions. The link between induction of EMT and the development of drug resistance has been confirmed for multiple cancer types and has gained the attention of researchers worldwide to identify new therapeutic targets that abrogate EMT and re-sensitize cancers to chemotherapeutic drugs. A recent report elegantly illustrates the mechanisms underlying EMT-dependent acquisition of resistance to diverse therapeutic agents observed in different types of cancers, and stratifies approaches for targeting the EMT program as a part of cancer treatment, focusing on prevention of EMT induction, selective targeting of cells that have undergone the EMT shift, and launching the reverse MET program in mesenchymal cancer cells [12].

In ovarian cancer, however, the association between EMT and drug resistance remains disputable, as numerous research data provide contradictory conclusions. For example, activation of Notch3 signaling in ovarian cancer OvCa429 cells (epithelial-type) launched EMT and attenuated carboplatin-induced apoptosis in these cells through inhibited ERK phosphorylation [247]. Similarly, promotion of EMT by hematopoietic PBX interacting protein [248], NANOG [249,250], TWIST1 [251,252], SNAI1 [253] FOXM1 [254] accompanied acquisition of ovarian cancer cell resistance to conventional therapeutic agents, such as cisplatin, carboplatin or paclitaxel. Contrary to these are data from a study that exploited sensitivity to cisplatin among 46 ovarian cancer cell lines and established a higher level of drug resistance in the cells with epithelial status [57]. Subsequent pathway analyses discovered activation of the NF-κB pathway by administered cisplatin exclusively in epithelial cells, resulting in defective apoptosis and cisplatin resistance [57]. Another study showed downregulation of miRNA-200 family members (microRNAs that act as supressors of EMT) in paclitaxel- and carboplatin-resistant ovarian cancer cells with a strong mesenchymal phenotype, but surprisingly, overexpression of miR-200c/miR-141 in these cells and partial restoration of epithelial traits led to a 6 − 8x higher resistance to carboplatin while showing no change in response to paclitaxel. Yet another study characterized tumor cell clusters from ovarian cancer patient ascites and identified that cells/clusters with mesenchymal markers were predominantly derived from chemo-naïve patients, whereas cellular components of the epithelial phenotype were found in ascites of patients with recurrent disease and chemoresistance [56]. Further, comparison of ascites-driven single cells of epithelial and mesenchymal morphology (confirmed by respective marker expression) depicted a significantly higher level of resistance to cisplatin in epithelial-type cells relative to mesenchymal-type cells [56].

Clearly, additional research is needed to fully understand the relationship between EMT/MET and chemoresistance in ovarian cancer, as it will allow recruitment of EMT regulators to combat EOCs significant challenges—multidrug resistance and metastatic aggressiveness. Given the distinct mechanism of ovarian cancer metastatic dissemination and the unique aspects of epithelial vs. mesenchymal EOC cells, nontrivial EMT-targeting therapeutic approaches might be required. In particular, current scientific knowledge highlights ovarian cancer epithelial-state cells as more chemo- and radiation resistant (as outlined in Section 3 and Section 5 of this commentary), raising a speculation that new therapeutic interventions may actually lie in EMT *promotion* for re-sensitization of EOC cells to therapeutic agents—a strategy opposite to that suggested for other cancer types [12]. However, pre-clinical data including those of our group (see Section 3 of the current review) indicate that acquisition of the mesenchymal phenotype in EOC is particularly associated with aggressive metastatic invasion. In this case, as our latest report concludes [45], targeting Ncad on the surface of mesenchymal-type EOC cells with Ncad-blocking peptides, such as the HAV-motif harboring drug ADH-1 (Exherin) or monoclonal antibodies may represent a promising anti-metastatic strategy. Future studies designed to resolve the EOC EMT/chemoresistance controversies and target the unique characteristics of EOC cells are warranted.

## 6. Computational Modeling Approaches to Understanding EMT/MET in EOC

Computational systems biology models have become an indispensable tool in analyzing highly empirical cancer progression data and can greatly contribute to elucidating the underlying principles of EMT/MET in EOC. Regulatory networks underlying these transitions in EOC as well as other cancer types involve multiple signaling pathways including TGF-β, EGF, HGF, FGF, NF-kB, Wnt, Notch, Hedgehog, JAK/STAT, Hippo [255], and hypoxia [256]. In addition, the mechanical properties of the extracellular matrix (ECM) such as density [257] and stiffness [258] also play role in EMT/MET. These signals trigger activation of EMT-inducing transcription factors involving ZEB1/2, SNAIL1/2, TWIST1, and Goosecoid, thereby repressing epithelial genes including Ecad. As mentioned previously, microRNA-mediated control of translation, splicing of mRNAs and epigenetic modifiers can also regulate EMT/MET [259,260]. Various feedback loops discussed can alter plasticity of the cell and enable the existence of intermediate phenotypes. Understanding how these multiple factors govern epithelial-hybrid-mesenchymal states stimulated the development of mathematical models to study the underlying mechanisms, as well as the dynamics, stability and reversibility of EMT. Although EOC-specific EMT/MET computational models are not well-represented in the literature, the existence of similar EMT/MET signaling pathways in different cancer types suggests logical extension of existent models to EOC.

### 6.1. Regulatory Networks-Based Models of EMT/MET

To delineate the emergent dynamics of EMT/MET regulatory networks, low- and high-dimensional kinetic models have been developed [261,262,263].

#### 6.1.1. Low-Dimensional Models

The two major low-dimensional models focus on describing individual reactions between a set of micro-RNAs families and comprise miR-34, miR-200 and EMT-TF ZEB and SNAIL players. As was reported recently [261,262] these networks allow for co-existence of epithelial (E) and mesenchymal (M) phenotypes along with a hybrid epithelial-mesenchymal (E-M) phenotype, observed experimentally in many studies revealing subpopulations of E, M, and E-M cells in various cell lines [80]. The fact that E-M clustering can result in a significantly larger amount of EOC secondary tumors as compared to pure E or M phenotype [81], therefore impacting metastatic success, makes the small-scale model a critical component in predicting the outcome of E, M and E-M cell interactions. The modeling approach developed by Lu et al. [261] uses a theoretical framework to account for microRNA- and transcription factor-mediated interactions. The model suggests that miR-200/ZEB feedback loop works as a switch allowing for three stable states and that hybrid E-M cells correspond to intermediate miR-200 and ZEB levels. In contrast, Tian et al. [262] proposed a simplified model applying mathematical forms to consider translational and transcriptional interactions. In their work, it is hypothesized that both miR-200/ZEB and miR-34/SNAIL act as bi-stable switches and the hybrid E-M phenotype is caused by low ZEB and high SNAIL levels.

The impact of other transcription factors modulating EMT/MET in the low-dimensional approach was also considered. In particular, GRHL2 and OVOL2 were shown to act as ‘phenotypic stability factors’ (PSFs) allowing for the existence of a hybrid E-M phenotype at a wider range of model parameters [72,264]. The regulatory network in the later study [264] coupled OVOL with miR-34/SNAIL and miR-200/ZEB circuits. The core of the EMT regulatory network comprised of self-inhibitory OVOL which formed a mutually inhibitory loop with ZEB and indirectly inhibited miR-200 via STAT3. TGF-β activated SNAIL, and BMP7/Smad4 pathway and C/EBP-β activated OVOL, whereas Wg signaling (Armadillo/dTCF) inhibited OVOL.

In application to ovarian cancer modeling, suppression of GRHL2 was recently shown to inhibit proliferation, invasion, and migration of ovarian cancer cells [265], emphasizing the importance of incorporating this factor into a low-dimensional EOC EMT/MET model. Additionally, extracellular communications such as those mediated by JAG1 were shown to be able to perform the role of PSF via Notch-Jagged signaling [266]. Furthermore, to quantify global stability of the hybrid phenotype in EOC EMT and transition dynamics among different phenotypes, the landscape and kinetic paths approach needs to be used to aid in understanding the mechanisms of EMT processes, and unveil possible roles for the intermediate E-M states [267,268].

#### 6.1.2. High-Dimensional Models

In contrast to low-dimensional kinetic models, high-dimensional, large-scale regulatory network models have been developed based on Boolean formalism focusing on the logical topology of the networks. Following this approach an EOC EMT network of multiple nodes and edges can be constructed to simulate the dynamics of transcription factors and microRNAs. This methodology has been successfully used in a study [263] that considered a network of 70 nodes and 135 edges to follow the dynamics of TGF-β-driven EMT in hepatocellular carcinoma. The nodes of the network represented molecular entities (proteins, small molecules, mRNAs) while the edges depicted activating and inhibitory relationships between nodes. Upstream signals (SHH, Wnt, HGF, PDGF, IGF1, EGF, FGR, Jagged, TGF-β, DELTA, CHD1L, Goosecoid, Hypoxia) regulated transcriptional regulators (SNAI1, SNAI2, FOXC2, TWIST1, ZEB1, ZEB2) through signal transduction pathways, which all converged on the regulation of E-cadherin state, defining the EMT output of the network. A related study [269] simulated multiple signaling pathways, microRNAs and transcription factors to derive mutations and functional changes in network nodes that result in altered metastatic behavior.

One of the advantages of using the logical network formalism is that it provides a larger set of steady states compared to kinetic-mechanism based models due to higher dimensionality of the system considered. The large scale network approach allows for better characterization of EMT by providing a more detailed profile of transient hybrid E-M states. Yet, Boolean modeling framework considers each node to take discrete ‘on’ and ‘off’ states which makes it difficult to simulate transitions involving continuous-like switches such as the transcription factor ZEB.

Overall, both low- and high-dimensional models complement each other in identifying regulatory network-based mechanisms of EMT/MET and can perform as an efficient computational tool to recognize stability factors allowing for existence of E-M hybrid phenotypes in EOC.

### 6.2. Omics-Based Models of EMT

Typical omics-level models of EMT use gene expression data which is then analyzed using statistical methods for identifying transition patterns and characterization of trajectories between phenotypes. By applying methods of chemical reaction dynamics, changes in transcriptional profile in the course of EMT were studied [270]. It was shown that a stable low-energy intermediate state exists in the landscape of the free energy changes during TGF-β1-induced EMT for the lung cancer cells coinciding with metabolic shifts. In another study [271], integration of time-course EMT transcriptomic data with public cistromic data allowed investigators to identify three synergistic master transcription factors, namely ETS2, HNF4A and JUNB, that regulated the transition between partial EMT states. Removal of these factors abrogated TGF-β-induced EMT. Likewise, application of these models to TGF-β-induced EMT in EOC can identify transcription factors responsible for the hybrid E-M phenotype which can be further examined experimentally.

Another useful model approach which was applied to EMT in EOC along with other cancer types was suggested in [272], enabling quantification of the EMT spectrum in the course of cancer progression. In this model, transcriptomics data are used to identify a generic EMT signature involving common molecular signatures of EMT across tumors and cell lines of different origins comprising bladder, breast, colorectal, gastric, lung and ovarian cancers. For a given sample the EMT score is then calculated using a two-sample Kolmogorov-Smirnov test in the range from −1 to +1 with the positive scores indicating mesenchymal, and negative scores corresponding to epithelial phenotype. Interestingly, the generic EMT scores of EOC tumors were slightly negative (around −0.1) pointing out a slight shift towards epithelial phenotype, whereas ovarian cancer cell lines revealed more mesenchymal phenotype with positive scores (+0.25). The results of the model suggest that EMT status does not necessarily translate to chemotherapeutic resistance and correlate with poorer survival.

### 6.3. Multiscale Models of EMT

The goal of multiscale models is to link the intracellular dynamics of EMT signaling pathways to the adhesion molecules on the cell surface and changes in tumor cell invasion through the extracellular matrix. A multiscale individual-based lattice-free model accounting for the intracellular dynamics of the Ecad-β-catenin interaction and external mechanical forces was developed [273]. Each cell was modeled as an isotropic elastic body permitting migration and division calibrated using cell-kinetic, biophysical and cell-biological experimental data. The results indicate that some of migratory features of EMT can be reproduced in the model when the extracellular gradient of chemoattractant is induced and the system of proteasomes responsible for degrading β-catenin is downregulated.

Monte Carlo-based model simulations of EMT were presented [274] with focus on the formation of cardiac cushions during embryonic development of the heart. Cell rearrangements are provided in terms of Steinberg’s differential adhesion hypothesis suggesting type-dependent adhesion along with motility levels sufficient to result in tissue conformations with the largest number of strong bonds. The model couples differential adhesion, EMT, cell proliferation and matrix production by mesenchymal cells, and predicts that increases in cell-ECM interactions might be more important in promoting EMT than decreases in cell-cell adhesion.

Stochastic and deterministic models describing tumor growth based on the cancer stem cell hypothesis in application to EMT were given [275], wherein the possibilities of using quantitative approaches for identification of an increase in stem cell activity following promotion of EMT were discussed. The cancer stem cell hypothesis implies that not all tumor cells are equal in terms of their roles in treatment resistance, therefore suggesting possible therapeutic targets in EOC. If EMT can lead to formation of CSCs from non-stem cancer cells, then EMT must be targeted along with CSCs, as non-stem population may recover the CSC pool.

Various computational and statistical models of EMT can contribute significantly by providing a quantitative assessment of EMT dynamics that incorporate EOC genetic and biophysical parameters, which can then be tested experimentally to yield further insight into mechanisms driving EMT.

## 7. Conclusions

Successful metastatic dissemination of EOC in the complex peritoneal microenvironment necessitates dynamic and reversible changes in cell-cell interaction as cancer progresses from the primary tumor to free-floating MCAs and matrix-anchored metastases. This unique metastatic niche provides a diversity of biochemical and biomechanical cues that, together with the inherent phenotypic plasticity of EOC cells, promotes EMT and MET at various stages in metastatic progression. Additional research designed to mechanistically integrate key drivers of the EMT/MET program will undoubtedly identify a wealth of potential therapeutic targets to inhibit successful metastatic spread and thereby improve survival of women with EOC.

## Figures and Tables

**Table 1:** Target and candidate genes with EMT-associated epigenetic modifications.

Target or Candidate Gene	Epigenetic Modification	Reference
CDH1 (Ecad), silenced	hypermethylation via 5′ CpG island	[129,130,131]
	methylation via ZEB1 through recruitment of DNMT1 to CDH1 promotor	[132]
	histone H3K27m3 demethylation at the SNAI1 promotor; mono-, di- and trimethylation of histones H3K36m2, H3K4m2, H3K9m3, H4K20m1, H3K9m1/2 by methyltransferases MMSET, LSD1, Suv39H1, SET8 and G9a at the TWIST, CDH1s and CDH2 promoters	[128,133,134,135,136]
CDH1 (Ecad), reactivated	deacetylation of SNAI2	[140]
SFRP5, silenced	hypermethylation in EOC through Wnt signaling pathway	[143]
IQGAP2, silenced	hypermethylated in EOC via Wnt/β-catenin signaling	[144]
CDH1, ERBB3, FGFBP1, IGFBP4, IL1RN, MMP9, SNAI3, SPP1, WNT11, WNT5B (downregulated)	TGF-β induced methylation	[142]
BMP1, COL1A2, COL3A1, COL5A2, FOXC2, GSC, KRT14, KRT7, MMP2, MMP3, RGS2, SNAI1, TCF4, TFPI2, TGFB2, WNT5A, ZEB2 (upregulated)	TGF-β induced methylation	[142]
